# Reduction in biogenic amines in douchi fermented by probiotic bacteria

**DOI:** 10.1371/journal.pone.0230916

**Published:** 2020-03-26

**Authors:** Fiona Long Yan Fong, Ka Yam Lam, Chun San Lau, Kin Hei Ho, Yeuk Hei Kan, Mui Yee Poon, Hani El-Nezami, Eric Tung Po Sze

**Affiliations:** 1 Department of Science and Environmental Studies, The Education University of Hong Kong, HKSAR, People’s Republic of China; 2 School of Science and Technology, The Open University of Hong Kong, HKSAR, People’s Republic of China; 3 School of Biological Sciences, The University of Hong Kong, HKSAR, People’s Republic of China; University of Nairobi, KENYA

## Abstract

Ecology studies showed that esophageal and gastric cancers are directly correlated with the consumption of processed foods. The carcinogenicity of traditional Chinese fermented foods such as douchi (fermented black beans or fermented black soybeans) is due to the presence of carcinogenic N-nitroso compounds, which are derived from biogenic amines. Among the various biogenic amines that can act as precursors of N-nitroso compounds, histamine and tyramine are considered to be the most toxic and are of public health concern when present in food. We have examined some douchi products on the market, and significant amounts of histamine and tyramine were found. The use of fermentation starters generated by subculturing fermented products with unknown microbiota would induce the risk of biogenic amines. As the microbiota used in fermentation is a crucial factor in determining the biogenic amines of fermented food, it is hypothesized that the possible harmful effects of douchi can be minimized through the use of fermentation starters composed of probiotic bacteria. This is the first study to investigate the potential of using probiotic bacteria in manufacturing douchi. *Lactobacillus rhamnosus* GG (LGG), *Lactobacillus casei* Shirota (LcS) and *Escherichia coli* Nissle 1917 (EcN) were used to ferment black beans in this study, and no tyramine was detected in black bean samples incubated with these three strains anaerobically at 37°C or 20°C. The starter culture strains, temperature and presence of oxygen during the incubation period were found to be critical to the generation of biogenic amines. The findings of this study can provide evidence-based insights and warrant further investigations on the potential of reducing the harmful compounds in food fermented with probiotic bacteria as well as the sensory evaluation of douchi fermented with probiotic bacteria.

## Introduction

Nasopharyngeal carcinoma (NPC) is a tumor arising from the epithelial cells lining the nasopharynx. The incidence and mortality rates of NPC are relatively low in western countries, yet it has been documented to be a common malignancy among Cantonese residing in Guangdong Province, including southern China and Hong Kong, followed by Taiwan, Malaysia, Vietnam, Guam and other areas [1]. NPC is, therefore, known as the “Canton tumour”. Similar to NPC, cancers of the upper gastrointestinal tract are prevalent and deadly in Asian countries. Proximal or distal gastric carcinoma (GC) and esophageal carcinoma (EC) are two other common cancers in Asian communities [2–5]. The high mortality rate and prevalence of NPC in Asian populations have raised interest in understanding the risk factors that lead to these cancers.

Epidemiological studies have indicated the extraordinarily high incidence of NPC, GC and EC in areas such as China and Hong Kong, implying the particular etiologies of these life-threatening cancers. Although human genetic variation may be one of the crucial factors for tumorigenesis [6], the special distribution of NPC and other cancers in the upper parts of the GI tract may also be attributed to the dietary habits inherent in traditional Chinese culture [7–9]. Douchi (fermented black beans or fermented black soybeans) is a traditional fermented food in China; douchi has been commonly consumed as a flavoring in Chinese cuisine for centuries and has been associated with NPC [10]. Etiological evidence has proven that the carcinogenicity and mutagenicity of processed foods is due to the presence of carcinogenic N-nitroso compounds [11, 12], such as nitrosamines and nitrosamides, the yields of which are enhanced by reacting with nitrate and nitrite, which are commonly found in salt and other sources for manufacturing processed foods, or by digestion in the stomach.

N-Nitroso compounds are formed by the decarboxylation of amino acids by bacterial enzymes [13–15]. N-Nitroso compounds are derived from biogenic amines that are ubiquitous in high-protein fermented foods such as douchi [16, 17]. It has also been used as a medicine for high blood pressure [18] and inflammatory disease (i.e., ‘heat’ syndrome in Chinese medicine) [19]. Among different biogenic amines, histamine and tyramine are considered to be the most toxic and are of public health concern [20–22]. These two biogenic amines have also been reported in various studies of traditional Chinese fermented foods. Food contaminated with substantial amounts of histamine may cause food intolerance in sensitive individuals and food poisoning [23], while food rich in tyramine may lead to hypertensive crises and, in a few cases, migraine [22, 24–26]. Nevertheless, although published information is limited, studies have shown that no adverse health effects occur in healthy individuals not consuming monoamino oxidase inhibitor (MAOI) drugs in food after exposure to 50 mg of histamine or 600 mg of tyramine [22, 23, 27].

The selection of microbes for fermentation starters used to produce bacteria-ripened foods is a crucial factor in determining the biogenic amines content in foods. However, regulation in the manufacturing processes and bacterial species used in fermentation starters in industry, especially small- to medium-sized traditional food manufacturing, is lacking. Different processors adopt different manufacturing methods, including processes and recipes [28, 29], and the microbial composition of the fermentation starter varies between batches and from the primitive starter [30]. The dynamic change in biogenic amine content may be due to variations in the microbial composition of fermentation starters through subsequent subculturing, together with other factors such as contamination in the uncontrolled environment. Measures to control the bacterial species used in the fermentation starter of douchi under a controlled environment are therefore believed to be of paramount importance to minimize or eliminate the harmful components in douchi. Studies have reported that the fermentation process of certain foods, such as soy sauce, involves the use of *koji* mold (e.g., *Aspergillus oryzae*), lactic acid bacteria (e.g., *Tetragenococcus halophilus*) and osmophilic yeasts (e.g., *Zygosaccharomyces rouxii*) [31–33]. Certain species of probiotic bacteria, such as lactobacilli [34], may be suitable for use in the fermentation process of douchi. Several studies have isolated and characterized lactic acid bacteria species from douchi samples on the market [35, 36], and some of these lactic acid bacteria may be probiotic species.

Probiotic bacteria are one of the “Generally Recognized As Safe (GRAS)” products recognized by the U.S. Food and Drug Administration (FDA) and are widely used as functional ingredients [37]. They are presumed to be beneficial and can prevent or delay the onset of certain cancers [38]. It is therefore hypothesized that the possible harmful effects of douchi can be minimized or even eliminated through the use of fermentation starters composed of potential probiotic bacteria. Two probiotic strains of lactobacilli and one probiotic strain of *Escherichia coli*, namely, *Lactobacillus rhamnosus* GG (LGG), *Lactobacillus casei* Shirota (LcS) and *E*. *coli* Nissle 1917 (EcN), were chosen as fermentation starters to ferment black beans in this study, as these strains have not been documented to produce biogenic amines in foods [22]. The amounts of histamine and tyramine were analyzed by lipid chromatography mass spectrometry. To the best of our knowledge, this is the first study to investigate the potential of using probiotic bacteria in manufacturing douchi and the harmful effects thereof.

## Material and methods

### Chemicals and reagents

Reference compounds of histamine and tyramine were purchased from Alfa Aesar, United Kingdom. HPLC-grade solvents of acetonitrile and acetone were obtained from Merck Millipore, MA, USA. Ultrapure water (<18.2 MΩ cm^-1^ resistivity) was prepared with a GenPure system (Thermo Scientific, MA, USA). All solvents were filtered through 0.22 μm filters before use.

### Preparation of starter culture from commercial douchi (Wild-type)

Four douchi samples of different brands were purchased from the local market for comparison and analysis. Douchi samples were stored at 4°C before the experiment. One of these commercial samples was used to prepare wild-type starter cultures for fermentation of black beans as a quality control. Douchi was homogenized with a stomacher. The supernatant was then added to lysogeny broth (LB) (Becton Dickinson, New Jersey, USA) and incubated at 37°C for bacterial growth [39].

### Preparation of starter cultures with probiotic bacteria

Three probiotic bacteria, namely, *Lactobacillus rhamnosus* GG (LGG; Valio Ltd, Helsinki, Finland), *Lactobacillus casei* Shirota (LcS; Yakult Honsha, Tokyo, Japan) and *E*. *coli* Nissle 1917 (EcN; Ardeypharm GmbH, Germany), were used to prepare starter cultures for fermentation of black beans. LGG and LcS were cultured in De Man, Rogosa and Sharpe (MRS) broth (LAB M Limited, Lancashire, UK), while EcN was cultured in LB. EcN is a gram-negative probiotic bacterium, whereas the two lactobacilli are gram-positive.

### Fermentation of black beans

The protocol for making douchi was designed with reference to the traditional method [36]. Raw black beans were purchased from a local market. They were autoclaved in deionized water to stew the beans and to ensure pure starter cultures during fermentation. Sterile black beans were inoculated with wild-type starter culture as well as starter cultures prepared with probiotic bacteria. Samples were incubated anaerobically or aerobically at 20°C or 37°C for three months. Samples harvested after incubation were stored at 4°C until the determination of biogenic amines. Negative control samples consisted of sterile black beans without the addition of any starter bacterial culture for incubation.

### Determination of biogenic amines

Fermented black beans and commercial douchi were homogenized with a stomacher. Two grams of the sample was mixed with 0.4 M perchloric acid (Sigma-Aldrich, St. Louis, MO, USA) and sonicated. The mixture was centrifuged at 1,620 xg for 10 minutes (Eppendorf 5804R). The supernatant was collected. The pellet was mixed with 10 mL of 0.4 M perchloric acid and centrifuged at 1,620 xg. The supernatant was collected. Two supernatants were pooled and filtered, and 0.5 M hydrochloric acid (VWR International, Pennsylvania, USA) was added to 25 mL; 250 μL of this mixture was adjusted with 2 M sodium hydroxide (Sigma-Aldrich, St. Louis, MO, USA) and mixed with saturated sodium bicarbonate solution (Sigma-Aldrich, St. Louis, MO, USA) and dansyl chloride solution (10 mg/mL) (Sigma-Aldrich, St. Louis, MO, USA). The mixture was incubated in the dark at 45°C for 1 hour. Twenty-five percent ammonium hydroxide (Sigma-Aldrich, St. Louis, MO, USA) was added to remove precipitates. Acetonitrile was added for analysis by liquid chromatography. Chromatography was conducted on a Waters ACQUITY UPLC@BEH C18 column 1.7μm 2.1 x 50 mm. A gradient elution of acetonitrile (100%) and acetonitrile (50%) with a flow rate of 0.2 mL/min, column temperature of 25°C and detection wavelength of 254 nm was used for separation. The levels of histamine and tyramine in samples were detected. The analytical method was validated. The limits of detection, quantification, linearity, precision and accuracy were determined.

## Results

### Biogenic amines in commercial douchi products

Of the four douchi products bought from the local market, histamine was detected in three (24.4–814.2 mg/kg), while tyramine was detected in all products (53.4–643.5 mg/kg) (Table 1).

**Table 1:** Biogenic amine levels in commercial douchi products.

Brand	Country of Origin	Histamine (mg/kg)	Tyramine (mg/kg)
Lee Kum Kee	Guangdong, China	74.6	53.4
Pearl River Bridge	China	814.2	643.5
Tung Chun	Hong Kong	24.4	106.5
Han River Bridge	China	ND	88.8

ND: none detected

### Appearance and texture of douchi

**Fig 1:**
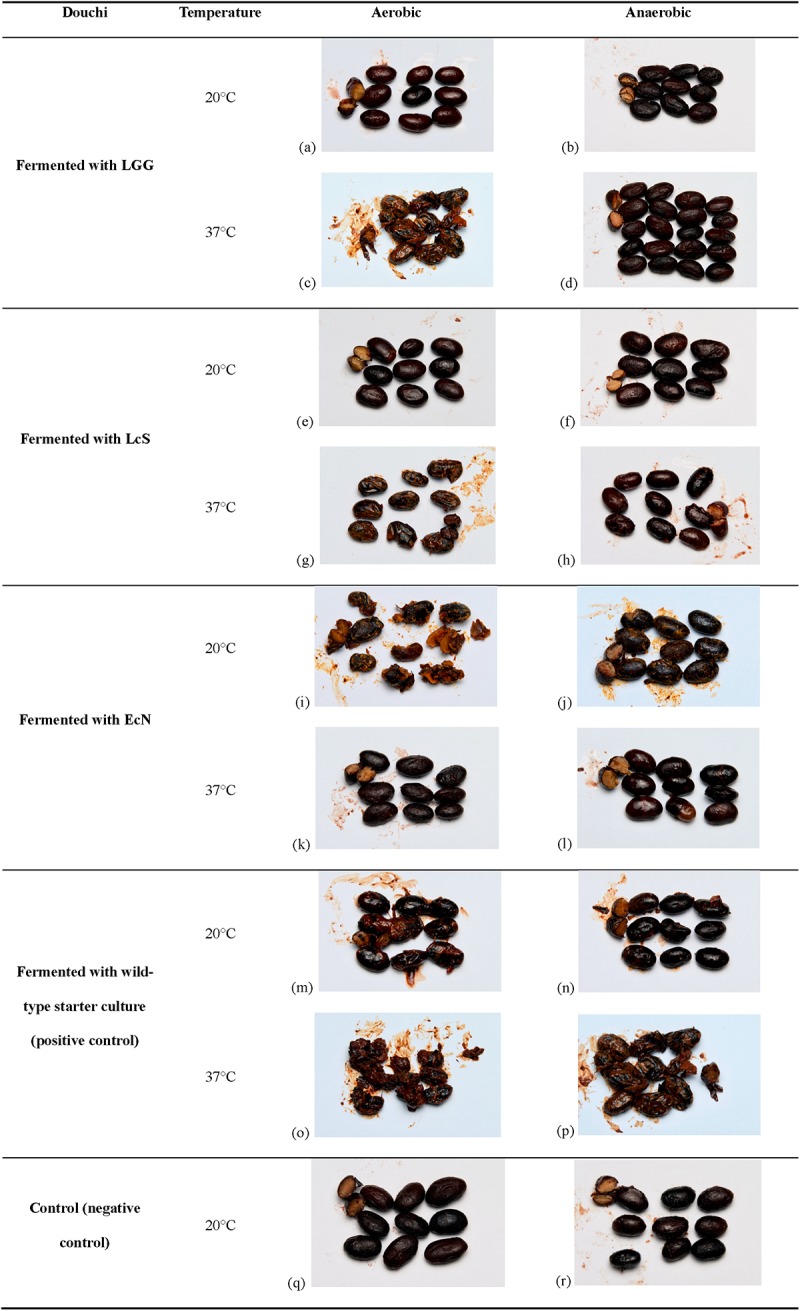
Appearance and texture of douchi fermented with probiotic bacteria and wild-type starter culture under different fermentation conditions. (a) Black beans fermented with LGG aerobically at 20°C; (b) Black beans fermented with LGG anaerobically at 20°C; (c) Black beans fermented with LGG aerobically at 37°C; (d) Black beans fermented with LGG anaerobically at 37°C; (e) Black beans fermented with LcS aerobically at 20°C; (f) Black beans fermented with LcS anaerobically at 20°C; (g) Black beans fermented with LcS aerobically at 37°C; (h) Black beans fermented with LcS anaerobically at 37°C; (i) Black beans fermented with EcN aerobically at 20°C; (j) Black beans fermented with EcN anaerobically at 20°C; (k) Black beans fermented with EcN aerobically at 37°C; (l) Black beans fermented with EcN anaerobically at 37°C; (m) Black beans fermented with wild-type starter culture aerobically at 20°C; (n) Black beans fermented with wild-type starter culture anaerobically at 20°C; (o) Black beans fermented with wild-type starter culture aerobically at 37°C; (p) Black beans fermented with wild-type starter culture anaerobically at 37°C; (q) Black beans with no bacteria added incubated aerobically at 20°C; (r) Black beans with no bacteria added incubated anaerobically at 20°C.

Due to the high level of histamine and tyramine detected in product B, as shown in Table 1, bacteria were isolated from this product for the preparation of a wild-type starter culture as a positive control. Black beans fermented with the wild-type starter culture or probiotic bacteria under different fermentation conditions resulted in douchi with different appearances and textures (Fig 1). Black beans that were fermented with LGG aerobically at 37°C, with EcN aerobically at 20°C and with the wild-type starter culture aerobically at 20°C and 37°C as well as anaerobically at 37°C were mushy, stringy and soggy. They also looked glazed, stale and brownish-yellow. Black beans that were fermented with LcS aerobically at 37°C, with EcN anaerobically at 20°C and with the wild-type starter culture anaerobically at 20°C were slightly mushy. The remaining eight douchi samples (black beans fermented with LGG and LcS aerobically and anaerobically at 20°C, black beans fermented with LGG and LcS anaerobically at 37°C, and black beans fermented with EcN aerobically and anaerobically at 37°C) were quite intact and looked similar to the negative control samples. The samples fermented with LcS and LGG smelled slightly sour.

### Biogenic amines level in douchi samples

The biogenic amine levels of the cultivated samples are summarized in Table 2, and no histamine was detected in any of the cultured samples. Tyramine was detected in all douchi samples fermented by the starter composed of bacterial culture isolated from commercial products (wild type, positive control), regardless of incubation conditions. For the douchi samples fermented with LGG, LcS and EcN and incubated anaerobically at 37°C and aerobically at 20°C, no tyramine was detected. However, when incubated at 37°C aerobically, 52.9, 82.4 and 62.0 mg/kg tyramine was detected in the douchi samples inoculated with LGG, LcS and EcN, respectively. In addition, 406.1 mg/kg tyramine was found in black beans fermented with EcN anaerobically at 20°C.

**Table 2:** Biogenic amine levels of douchi fermented with probiotic bacteria and wild-type starter culture.

Douchi	Temperature	Condition	Histamine (mg/kg)	Tyramine (mg/kg)
**Fermented with LGG**	20°C	Aerobic	-	-
		Anaerobic	-	-
	37°C	Aerobic	-	52.9
		Anaerobic	-	-
**Fermented with LcS**	20°C	Aerobic	-	-
		Anaerobic	-	-
	37°C	Aerobic	-	82.4
		Anaerobic	-	-
**Fermented with EcN**	20°C	Aerobic	-	-
		Anaerobic	-	406.1
	37°C	Aerobic	-	62.0
		Anaerobic	-	-
**Fermented with wild-type starter culture (positive control)**	20°C	Aerobic	-	86.5
		Anaerobic	-	132.4
	37°C	Aerobic	-	203.0
		Anaerobic	-	30.9
**Negative control**	20°C	Aerobic	-	-
		Anaerobic	-	-

"–" denotes not detected or below limit of detection (LOD)

## Discussion

This is a novel study to investigate the potential of using probiotic bacteria in manufacturing douchi and the harmful effects thereof. In this study, autoclaved black beans were fermented with different probiotic bacteria and bacteria isolated from a commercial douchi product (wild type, positive control). The results showed that the appearance, texture and odor varied among samples. For the same bacterial species, fermentation conditions such as aerobic conditions and temperature conferred different flavor characteristics to black beans. Our results were consistent with those of Hu et al. (2010). Hu et al. (2010) showed that the duration of fermentation influenced the firmness of black soybeans inoculated with *Bacillus natto* [40]. The firmness of the fermented black soybeans decreased with the time of fermentation, and the firmness of the fermented beans was significantly different from that of the unfermented beans after 48 hours of fermentation [40]. Changes in flavor characteristics by fermentation conditions were also observed and reported in other foods. In addition to the fermentation conditions, the fermentation of the black beans could be affected by the bacterial species. In general, the appearance, texture and odor of the black beans fermented with LcS and LGG were similar to those of the commercial douchi products in the study. Yang et al. (2019) showed that microbial variability determines the favors of douchi after fermentation [41].

The levels of biogenic amines in the four commercially available douchi products ranged from 24.4 mg/kg to 814.2 mg/kg (average: 304.4 mg/kg) for histamine and from 53.4 mg/kg to 643.5 mg/kg (average: 223.1 mg/kg) for tyramine in the study (Table 1). Tsai et al. (2007) analyzed commercial douchi products, and obtained an average histamine level of 290 mg/kg [16], whereas Li et al. (2018) detected histamine levels of only up to 213 mg/kg in douchi products [17]. In addition, Li et al. (2018) found tyramine in 14 of 15 douchi samples with levels ranging from 1.37 mg/kg to 64.33 mg/kg [17]. The bacteria (i.e., wild type; positive control) were isolated from the commercial product to ferment black beans in the study, and substantial tyramine (from 30.9 to 203 mg/kg) was detected in all fermentation conditions. Starters made by subculturing fermented products under uncontrolled conditions with unknown species would induce the risk of biogenic amines. The discrepancy in the concentration of biogenic amines in douchi with comparable microbiota may be due to the complex interaction of factors involved in the production of biogenic amines, as suggested by Jairath, Singh et al. (2015) [42]. For the black beans fermented with probiotic bacteria in the study, no biogenic amines were detected in the majority of the samples, particularly those fermented with LGG or LcS under anaerobic conditions. The findings support that probiotic bacteria, especially *Lactobacillus* species, may be potential candidates in the fermentation of douchi and other traditional Chinese foods.

## Conclusions

In conclusion, the study demonstrates that the biogenic amine levels, and hence the possible harmful effects of douchi, can be minimized or even eliminated through the use of fermentation starters composed of probiotic bacteria such as LGG, LcS and EcN; for the black bean samples incubated with these three strains anaerobically at 37°C and aerobically at 20°C, no tyramine was detected. The starter culture strains, temperature and presence of oxygen during incubation were found to be critical to the generation of biogenic amines. The findings of this study should aid similar studies of other Chinese fermented foods in the future. They provide evidence-based insights and warrant further *in vitro* and *in vivo* investigations on the potential of reducing the harmful effects in food fermented with probiotic bacteria as well as sensory evaluation on douchi fermented with probiotic bacteria.
